# Functional Characterization and Potential Regulatory Role of *MdWRKY31* in Cold Tolerance

**DOI:** 10.3390/ijms27125560

**Published:** 2026-06-19

**Authors:** Yonghui Liang, Guishuang Wang, Xiaomeng Yang, Bowen Zhang, Yuting Ma, Yujie Ji, Deguo Han

**Affiliations:** 1 The Key Laboratory of Biology and Genetic Improvement of Horticultural Crops (Northeast Region), Ministry of Agriculture and Rural Affairs/National-Local Joint Engineering Research Center for Development and Utilization of Small Fruits in Cold Regions, College of Horticulture, Northeast Agricultural University, Harbin 150030, China; 2 State Key Laboratory of Crop Stress Biology for Arid Areas/Shaanxi Key Laboratory of Apple, College of Horticulture, Northwest A&F University, Yangling 712100, China

**Keywords:** *Malus* × *domestica*, WRKY31, cold tolerance, antioxidant defense system

## Abstract

Identifying cold-resistance genes is essential for improving the ability of apples (*Malus* × *domestica*) to tolerate low temperatures, as cold stress significantly limits their growth and productivity. The *MdWRKY31* gene was cloned from apple, and its sequence characteristics, expression pattern, and biological function were systematically investigated. Bioinformatic analysis indicated that *MdWRKY31* belongs to the group II WRKY transcription factors and is localized in the nucleus. Expression analysis revealed that *MdWRKY31* transcript levels were markedly upregulated under low-temperature stress. To further explore its function, *MdWRKY31* was heterologously overexpressed in tomato (*Solanum lycopersicum*). Following low-temperature treatment, transgenic tomato plants exhibited significantly reduced accumulation of reactive oxygen species, markedly enhanced activities of antioxidant enzymes (SOD, POD, and CAT), increased contents of proline and soluble protein, and a notable decrease in malondialdehyde levels. Additionally, transcript levels of *SlCBF1*, *SlCBF2*, *SlCBF3*, *SlICE1*, along with the ABA signaling-related genes *SlNCED1* and *SlABI5*, were markedly elevated. Further molecular docking showed that the MdWRKY31 protein has strong binding affinity to the W-box elements in the promoters of *SlCBF1* suggesting that it may regulate the expression of these genes through direct protein–DNA interactions. These findings indicate that *MdWRKY31* improves plant cold tolerance by CBF-dependent pathways to modulate antioxidant defenses and osmotic balance. These findings identify candidate genetic resources for breeding cold-resistant apple cultivation.

## 1. Introduction

Drought, salinity, extreme temperatures, UV radiation, and heavy metals are major abiotic constraints that limit plant growth and development. Occurring alone or in combination, they disrupt physiological and molecular processes, endangering global agricultural productivity and ecosystem stability [1,2]. Among abiotic stressors, low temperature is a key limiting factor for plant geographic distribution, directly impacting growth, development, and metabolic function [3,4,5]. Through long-term natural selection, plants have evolved sophisticated regulatory mechanisms to respond to low-temperature stress, encompassing the perception of cold signals, signal transduction, and the coordinated regulation of downstream physiological and biochemical responses. Under moderate low-temperature conditions that do not cause freezing injury, plants can undergo a cold acclimation process, which effectively mitigates the adverse effects of cold environments and enhances their tolerance to low-temperature stress [6,7]. Therefore, deciphering the physiological and molecular processes of cold acclimation, together with the functional characterization of cold-tolerance genes and low-temperature signaling pathways, is crucial for advancing our understanding of plant cold adaptation.

Low-temperature stress-induced morphological changes are direct manifestations of plant injury, typically including leaf curling, wilting, and chlorosis, and in severe cases, tissue necrosis [8]. These phenotypic alterations are closely associated with compromised cellular architecture and function, among which disruption of cell membrane integrity is considered a key physiological basis of low-temperature injury. Biological membranes not only serve as the primary barrier protecting cells from environmental fluctuations but also play central roles in material transport and signal transduction, making them critical sites for the perception of low-temperature stress [9]. Under low-temperature conditions, the plasma membrane is usually the first cellular component to be affected, and membrane damage is a widely adopted indicator reflecting the severity of low-temperature stress [10]. Low temperature can induce membrane lipid peroxidation, reducing membrane structural stability and selective permeability, thereby causing leakage of cellular contents and increased electrolyte conductivity in the surrounding medium [11,12]. Soluble sugars and proline, as low-molecular-weight osmolytes, help maintain osmotic balance under low-temperature conditions [4]. Plants actively accumulate these osmolytes to enhance cellular osmotic potential, thereby improving water absorption capacity and maintaining cellular structural stability and basic metabolic activities [13]. In addition, low-temperature stress typically triggers substantial accumulation of reactive oxygen species (ROS), which triggers oxidative stress and leads to damage of cellular macromolecules, including proteins, lipids, and nucleic acids [14,15,16].

Transcription factors (TFs) are pivotal in controlling gene expression and signal transduction during plant stress responses by binding to cis-acting elements and modulating target gene transcription [17]. Among the multiple TF families involved in stress adaptation, such as WRKY, NAC, bZIP, AP2/ERF, and MYB, the WRKY family is the most extensively studied [18,19,20]. WRKY transcription factors regulate gene expression by specifically recognizing W-box elements in target gene promoters [21,22]. The WRKY domain, approximately 60 amino acids in length, contains a highly conserved WRKYGQK motif along with a zinc-finger structure, and WRKY proteins are divided into three groups according to the number of WRKY domains and the type of zinc-finger motif [22,23]. WRKY transcription factors are key regulators of plant growth and development, and are also critically involved in stress responses, particularly to cold stress. For example, AtWRKY34 negatively regulates the CBF-mediated cold response and affects pollen development [24]. OsWRKY63 negatively regulates cold tolerance in rice (*Oryza sativa*) by repressing OsWRKY76 and other genes involved in cold response and ROS scavenging [25]. In watermelon (*Citrullus lanatus*), overexpression of ClWRKY20 enhances cold tolerance in transgenic *Arabidopsis thaliana* by alleviating oxidative stress, reducing membrane damage, and increasing proline accumulation [26]. In grapevine (*Vitis vinifera*), both VaWRKY12 and VaWRKY33 positively confer to cold tolerance in grape callus [27,28]. Similarly, PpWRKY18 improves cold tolerance by activating the expression of *PpPOD41* and improving antioxidant enzyme activity [29]. WRKY transcription factors are key players in plant adaptation to low-temperature stress. By regulating redox homeostasis, osmotic adjustment, and hormone signaling pathways, they are critical to cold tolerance and hold promise for the genetic improvement of cold-resistant crops.

Apple (*Malus* × *domestica*) is a widely cultivated fruit tree valued for its crisp texture and high nutritional value. However, climate change has led to more frequent abiotic stresses, such as drought, salinity, low temperature, and osmotic stress, which severely impair apple growth, fruit quality, and yield, limiting sustainable orchard production [30]. In Northeast China, low temperatures and prolonged cold periods are particularly prominent, with cold stress being especially significant. Prolonged low temperatures impair apple tree growth and metabolism, frequently resulting in severe yield losses or complete crop failure [31]. Accordingly, investigating the molecular pathways governing apple’s response to abiotic stresses, especially low temperature stress, is crucial for enhancing its environmental adaptability and promoting the sustainable growth of the industry.

WRKY transcription factors are central to apple adaptation under abiotic stress conditions. *MdWRKY30* confers tolerance in transgenic apple callus and other plant materials via positive regulation of osmotic and salinity stress responses [32]. Similarly, MdWRKY115 mediates positive regulation of drought and osmotic stress, and its overexpression in *Arabidopsis* and apple significantly improves tolerance to these stresses [33]. In addition to salt and water stress, WRKY transcription factors also contribute to apple’s response to heavy metal stress. For example, MdWRKY11 binds to the promoter region of the copper transporter gene *MdHMA*5, thereby modulating its expression and enhancing apple tolerance to copper stress [34]. Overall, WRKY transcription factors generally positively modulate apple’s responses to multiple abiotic stresses, however, their roles and molecular mechanisms under low-temperature stress remain largely unknown and warrant further investigation.

Previous studies have shown that the WRKY31 homolog proteins are involved in the responses of various plant species to abiotic stresses, including cold stress and drought stress. For example, the transcription factor TaWRKY31 in wheat participates in regulating plant tolerance to drought stress [35]. CiWRKY31 is one of the cold-induced WRKY transcription factors and plays a positive role in the regulation of cold tolerance [36]. PoWRKY31 directly binds to the promoter of PoWRKY75 and activates its expression, which subsequently induces the expression of PoCCoAOMT [37]. This leads to increased lignin accumulation, improved water retention capacity, and enhanced reactive oxygen species (ROS) scavenging ability under drought stress, thereby improving plant drought tolerance.

Here, *MdWRKY31* was isolated from apple and shown to be highly induced by low-temperature stress. By transforming this gene into tomato and combining phenotypic observations and physiological measurements under low temperature, we systematically analyzed *MdWRKY31′*s role in regulating plant responses to low-temperature stress. These results reveal new molecular insights into WRKY transcription factors’ role in apple’s response to low-temperature stress.

## 2. Results

### 2.1. Molecular Characterization and Low-Temperature-Induced Expression Analysis of MdWRKY31

**Figure 1:**
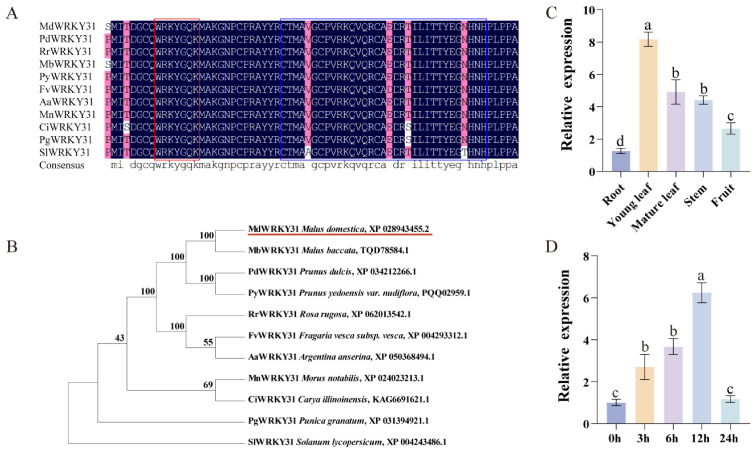
Molecular characterization and low-temperature-induced expression analysis of *MdWRKY31*. (**A**) Alignment analysis of the amino acid sequences of *MdWRKY31* and WRKY proteins from other species. The conserved WRKY amino acid motif is highlighted in a red box; the WRKY zinc finger motif is highlighted in a blue box; (**B**) Phylogenetic tree of WRKY31 proteins from different species; (**C**) Relative expression levels of *MdWRKY31* in different tissues; (**D**) Expression analysis of *MdWRKY31* under cold stress at 0 h, 3 h, 6 h, 12 h, and 24 h. Different lowercase letters (e.g., a, b, c, d) indicate significant differences at the *p* < 0.05 level.

*MdWRKY31* is 1836 bp in length, encoding a 611-amino-acid protein with a WRKY domain and a C2H2-type zinc-binding motif (C-X5-C-X23-HXH), features typical of class II WRKY transcription factors (Figure S1 and Figure 1A). The MdWRKY31 protein sequence was aligned with WRKY31 proteins from other species, and a phylogenetic tree was subsequently generated using MEGA software (Figure 1A,B). The results showed that MdWRKY31 had the highest homology with MbWRKY31 (*Malus baccata*, TQD78584.1) from the Rosaceae family, and grouped into a single clade in the phylogenetic analysis. In contrast, SlWRKY31 from tomato was located in a more distant branch, suggesting a relatively distant evolutionary relationship between apple and tomato WRKY31 proteins. SOPMA analysis indicated that the secondary structure of MdWRKY31 consists of 20.46% α-helix, 72.67% random coil, and 6.87% extended chain and tertiary structure prediction further showed that the protein contains α-helices (blue), extended chains, and random coils (orange) (Figure S1).

The qRT-PCR analysis revealed the expression profiles of *MdWRKY31* across different apple tissues. Expression was predominant in leaves, intermediate in stems and fruits, and minimal in roots (Figure 1C). To evaluate its response pattern to low temperature, newly emerged leaves were treated at 4 °C and the transcription levels of *MdWRKY31* were measured at 0, 3, 6, 12, and 24 h after treatment (Figure 1D). The expression level reached the peak at 12 h of low-temperature treatment, which was approximately 5.7 times that of the control group. Collectively, *MdWRKY31* likely plays a pivotal role in plant cold stress responses.

### 2.2. Subcellular Localization of the MdWRKY31 Protein

**Figure 2:**
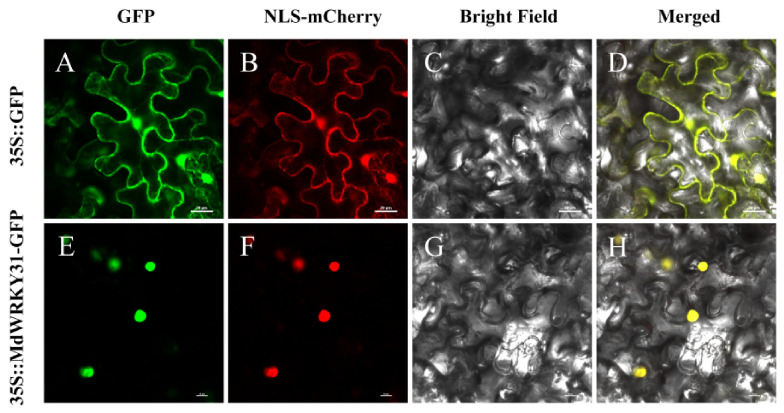
Subcellular localization analysis of MdWRKY31 protein. (**A**–**D**) Fluorescence distribution of the control vector 35S::GFP in tobacco leaf epidermal cells. GFP fluorescence (green) is widely distributed in the cytoplasm and cell membrane, NLS-mCherry nuclear marker (red) labels the nucleus, and bright-field and merged images show the intact cell morphology. (**E**–**H**) Fluorescence distribution of 35S::MdWRKY31-GFP fusion protein in tobacco leaf epidermal cells. GFP fluorescence (green) is concentrated only in the nucleus, completely overlapping with the NLS-mCherry nuclear marker (red), indicating that MdWRKY31 protein is localized in the nucleus. Bar: 20 μm.

To further characterize the function of MdWRKY31, we constructed the pCAMBIA1300-*MdWRKY31*::GFP expression vector and used pCAMBIA1300::GFP as a control. Each vector was transformed into tobacco leaf epidermal cells via Agrobacterium, and GFP fluorescence was examined under confocal microscopy. The control vector pCAMBIA1300::GFP displayed uniform green fluorescence throughout the cells, while pCAMBIA1300-*MdWRKY31*::GFP fluorescence was restricted to the nucleus (Figure 2). These results support that MdWRKY31 is a nuclear protein likely functioning as a transcriptional regulator.

### 2.3. MdWRKY31-OE Enhances the Cold Tolerance of Tomatoes

**Figure 3:**
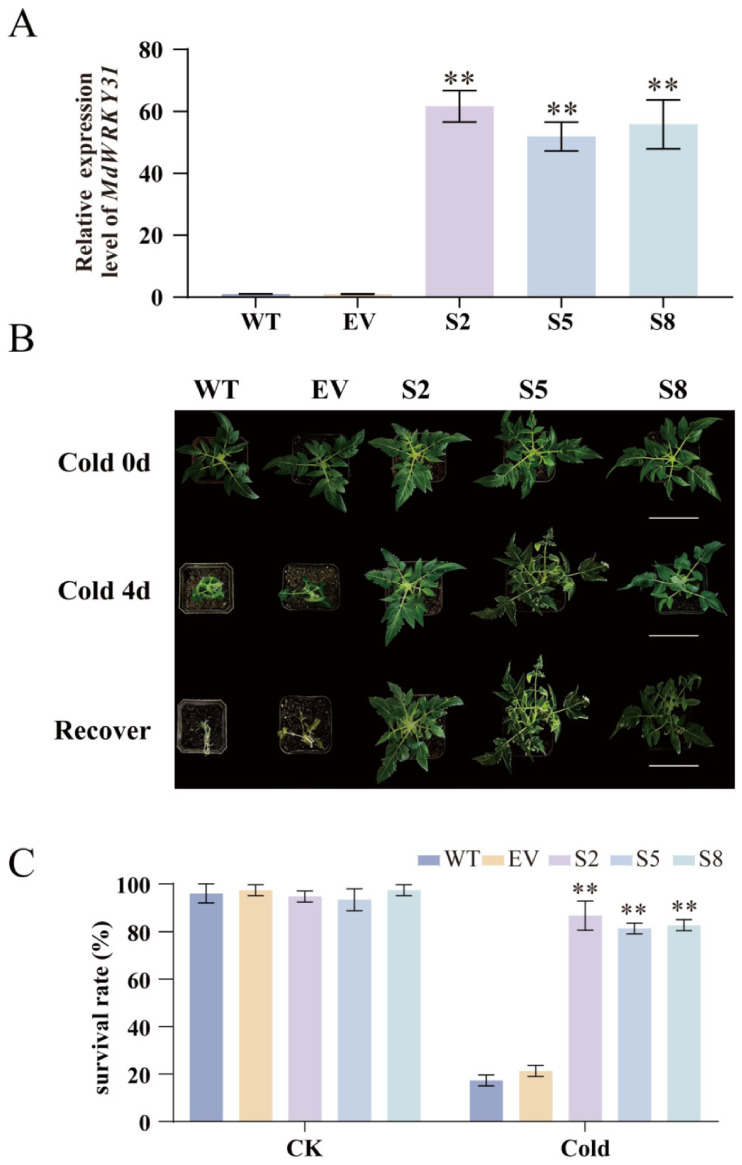
*MdWRKY31* overexpression enhances cold tolerance. (**A**) Relative expression levels of *MdWRKY31* in WT, EV, and overexpression lines (S2, S5, S8); (**B**) Phenotypes of control and transgenic lines under cold stress and recovery. Bar: 3 cm; (**C**) Survival rates of each line under control (CK) and cold treatment. The survival rate was determined from three independent biological replicates, each consisting of 25 plants. ** indicate significant (*p* < 0.05) and extremely significant (*p* < 0.01) differences, respectively.

We constructed the *MdWRKY31* overexpression vector and successfully transformed it into tomatoes (S1, S2, S3, S4, S5, S6, S7, S8) to explore the function of *MdWRKY31* in cold resistance (Figure S2). S2, S5, and S8 were chosen for further experiments due to their significantly higher expression levels (Figure 3A). All tomato lines displayed similar phenotypes under normal growth conditions (Figure 3B). Following 4 days at 4 °C, WT and EV plants displayed severe leaf wilting, whereas the three high-expression lines (S2, S5, S8) remained largely unaffected. After 7 days of recovery, only a few transgenic plants showed slight chlorosis, whereas WT and EV leaves were completely withered (Figure 3B). After cold stress, WT and EV plants showed survival rates of 17.33% and 21.33%, respectively, while S2, S5, and S8 lines achieved 86.67%, 81.33%, and 82.67% (Figure 3C). Therefore, overexpression of MdWRKY31 markedly enhances cold tolerance in tomato plants.

### 2.4. Analysis of Enhanced Reactive Oxygen Species (ROS) Scavenging Capacity in MdWRKY31-OE Tomato Under Cold Stress

**Figure 4:**
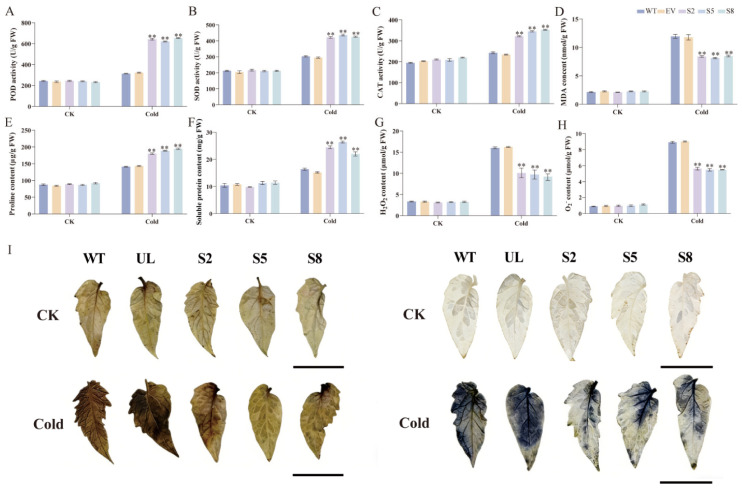
Antioxidant and physiological responses of MdWRKY31-overexpressing plants to low-temperature stress. (**A**) Peroxidase (POD) activity; (**B**) Superoxide dismutase (SOD) activity; (**C**) Catalase (CAT) activity; (**D**) Malondialdehyde (MDA) content; (**E**) Proline content; (**F**) Soluble protein content; (**G**) Hydrogen peroxide (H_2_O_2_) content; (**H**) Superoxide anion (O_2_^−^) content. CK: Control treatment; Cold: Low temperature treatment; (**I**) NBT and DAB staining analyses of leaves from MdWRKY31 overexpression plants under low temperature stress; Upper row shows DAB staining (detecting H_2_O_2_ accumulation, brown), and lower row shows NBT staining (detecting O_2_^−^ accumulation, blue–purple). All data were obtained from three independent biological replicate experiments. Bar: 1 cm. ** Indicates extremely significant difference (*p* < 0.01).

Physiological indicators were examined under normal and cold stress conditions in WT, EV, and T2 transgenic lines (S2, S5, S8) (Figure 4A–H). Physiological indicators were similar across all groups prior to treatment. All groups showed similar physiological indicators prior to treatment. After low-temperature stress, the transgenic lines accumulated more proline, soluble protein, and antioxidant enzymes (CAT, SOD, POD), but less MDA, H_2_O_2_, and O_2_^−^ than the WT and EV controls.

H_2_O_2_ and O_2_^−^ levels in leaves of WT, EV, and T2 transgenic lines (S2, S5, S8) were assessed by DAB and NBT staining, respectively (Figure 4I). Under control (CK), all groups exhibited similar leaf staining, showing light yellow or white coloration. Under cold stress, WT and EV leaves showed strong DAB and NBT staining, indicating high ROS accumulation, whereas transgenic lines exhibited weak staining (Figure 4I). In conclusion, overexpression of *MdWRKY31* improved tomato cold tolerance by enhancing antioxidant defense and alleviating oxidative injury.

### 2.5. Cold-Responsive Gene Expression in MdWRKY31-OE Tomato Under Low-Temperature Stress

**Figure 5:**
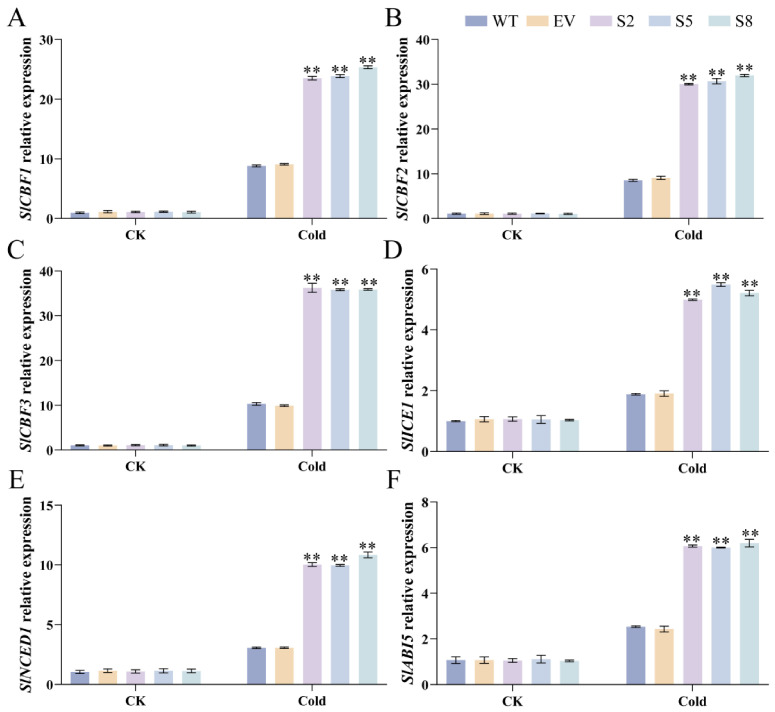
Expression of cold-responsive genes in *MdWRKY31*-overexpressing plants under low-temperature stress. (**A**–**F**) Relative expression levels of *SlCBF1*, *SlCBF2*, *SlCBF3*, *SlICE1*, *SlNCED1*, and *SlABI5* genes in each line after cold treatment, respectively; CK: Control treatment; Cold: Low temperature treatment; ** indicates extremely significant difference (*p* < 0.01).

To analyze the molecular regulatory mechanism of MdWRKY31 in enhancing the cold tolerance of transgenic plants, we quantified the transcript abundance of multiple cold-responsive genes, including *SlCBF1*, *SlCBF2*, *SlCBF3*, *SlICE1*, *SlNCED1*, and *SlABI5*. Low-temperature stress induced these genes in WT, EV, and *MdWRKY31*-OE lines, but transgenic tomato plants exhibited significantly higher expression levels than WT and EV (Figure 5). *MdWRKY31* overexpression potently activated cold stress signaling. The CBF-ICE module appears to be activated by this factor. Such activation likely contributes to improved cold tolerance in transgenic plants, promoting ABA biosynthesis and signaling, and inducing downstream cold-responsive genes.

### 2.6. Promoter Cis-Acting Element Distribution and Molecular Docking Analysis

**Figure 6:**
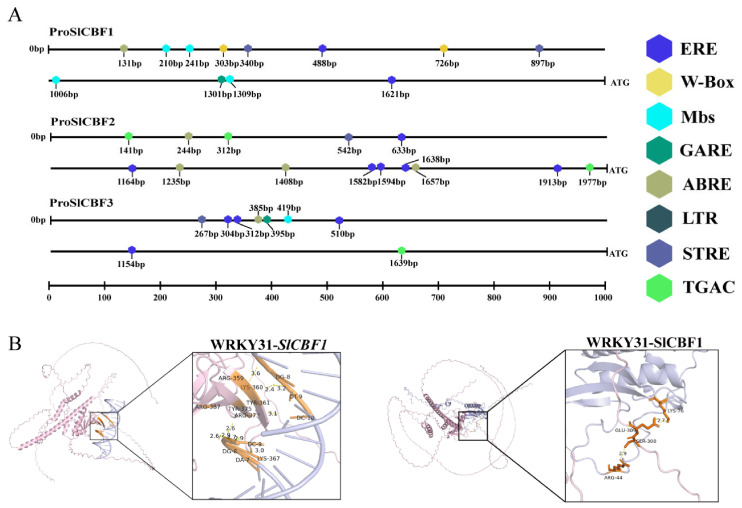
Promoter cis-acting element distribution and molecular docking analysis. (**A**) The structural features of the ProSICBF1, ProSICBF2, ProSICBF3 promoters; (**B**) Molecular docking analysis of *MdWRKY31* with downstream target genes analysis.

To analyze whether *MdWRKY31* participates in apple plant low-temperature response through CBF-dependent or CBF-independent pathways, promoter structural of *SlCBF1*, *SlCBF2*, and *SlCBF3* were analyzed, which revealed that promoter regions of *SlCBF1* contain W-box cis-acting element, the binding site of WRKY transcription factors (Figure 6A). Molecular docking results showed that the MdWRKY31 protein can form a stable three-dimensional interaction complex with the W-box elements in the promoter regions of *SlCBF1* (Figure 6B). We also predicted a docking model between the MdWRKY31 and SlCBF1 proteins, and the binding analysis showed that stable interaction forces also exist between the two proteins. The binding sites are mainly located within functional domains and are stabilized through hydrogen bonding and hydrophobic interactions. These findings suggest that WRKY31 may participate in the regulation of plant stress response signaling networks by directly regulating *SlCBF1*, a key factor in the CBF pathway.

## 3. Discussion

Low-temperature stress significantly restricts crop yield and quality, and plants enhance their adaptation to such stresses through complex signaling networks [38,39]. WRKY transcription factors are key transcriptional regulators that orchestrate plant responses to diverse abiotic stresses. Although WRKY transcription factors have been implicated in stress tolerance in *Malus* × *domestica* and *Malus baccata*, their function in apple cold tolerance mechanisms remains to be further explored [33,40,41]. A new WRKY IIb subfamily member was identified and subsequently cloned in this study, *MdWRKY3*1, and systematically analyzed its role in low temperature tolerance. MdWRKY31 contains a conserved WRKY domain and a C2H2-type zinc finger motif, characteristic features of WRKY transcription factors. Phylogenetic analysis revealed that MdWRKY31 exhibits high sequence similarity to MbWRKY31 from *Malus baccata*. MdWRKY31 localizes to the nucleus, consistent with the nuclear localization pattern of other WRKY transcription factors [42,43].

Expression pattern analysis revealed that *MdWRKY31* is expressed across various tissues in *Malus* × *domestica*, with markedly higher transcript levels in mature and young leaves than in roots, and the highest expression detected in young leaves. Notably, its expression peaked at 12 h of low temperature treatment, *MdWRKY31* likely serves as an early-responsive transcription factor in the cold signaling pathway. This expression pattern mirrors that of *AtWRKY34* and *OsWRKY63* under cold stress, implying conserved WRKY functions under cold stress [24,25]. To clarify the function of MdWRKY31, it was heterologously expressed in tomato plants. Heterologous expression in tomato has become a convenient and widely accepted approach for investigating the biological functions of apple genes. For example, heterologous overexpression of the apple gene *MdWAPL1* promotes fruit morphogenesis and alters fruit quality in tomato, while *MdSWEET23* affects sugar metabolism and enhances cold tolerance in tomato [44,45]. When exposed to cold conditions, the transgenic plants exhibited markedly improved tolerance, as indicated by reduced injury and increased survival rates. This finding is consistent with the reported functions of CiWRKY31 and KoWRKY40 in cold stress response [36,46].

Low-temperature stress promotes ROS overaccumulation, resulting in oxidative damage. In response, plants enhance antioxidant defenses to detoxify ROS and maintain redox homeostasis, with peroxidase activity representing a key marker of scavenging efficiency [47,48]. Under cold stress conditions, the activities of SOD, POD, and CAT were significantly elevated in *MdWRKY31*-OE tomato lines compared with WT plants, while NBT and DAB staining were substantially reduced, suggesting decreased levels of O_2_^-^ and H_2_O_2_. These results suggest that MdWRKY31 may positively regulate the antioxidant defense system, improving ROS scavenging ability and thereby increasing plant tolerance to cold stress. In addition to inducing oxidative damage, cold stress also leads to cellular osmotic imbalance and membrane injury. The maintenance of intracellular osmotic stability relies significantly on osmoprotective substances, notably proline and soluble proteins [49,50]. Malondialdehyde (MDA), generated during membrane lipid oxidation, is frequently employed to assess oxidative damage and its consequences for cellular integrity [51]. Under cold stress, *MdWRKY31*-OE lines accumulated more proline and soluble proteins but less MDA, suggesting enhanced osmotic regulation and reduced lipid peroxidation, thus improving cold tolerance.

The CE1-CBF-COR pathway is a key regulatory network in cold acclimation [52]. Cold-induced CBF transcription factors activate downstream cold-responsive genes, and their expression is modulated by various upstream regulators that influence cold tolerance [53,54,55]. In the present study, cold treatment markedly increased the transcript levels of *SlCBF1*, *SlCBF2*, and *SlCBF3* in transgenic tomato plants compared with WT lines, suggesting that *MdWRKY31* promotes cold tolerance through CBF-dependent signaling [56,57]. Previous studies have demonstrated that ICE1 is an upstream regulator of the CBF signaling pathway. Although the transcriptional level of *SlICE1* is not necessarily induced by low temperature, ICE1 positively regulates cold tolerance through the activation of downstream *CBF* genes. Consistent with the findings of Chinnusamy et al., the elevated expression of cold-responsive genes observed in *MdWRKY31*-overexpressing plants suggests that *MdWRKY31* may be involved in the regulation of the ICE1–CBF cold-response pathway [57]. This observation is consistent with previous findings in tomato. In addition to the CBF pathway, abscisic acid (ABA) is a critical regulator of plant responses to low-temperature stress, plants can improve stress tolerance via ABA-dependent mechanisms, including the induction of ABA-responsive genes such as *RD29B* and *RD22* [58,59,60]. In agreement with this, transcript levels of *SlNCED1* and *SlABI5* were markedly elevated in transgenic lines upon cold exposure, suggesting that *MdWRKY31* may also contribute to cold tolerance by participating in the ABA-mediated signaling pathway. However, whether *MdWRKY31* directly regulates these genes or operates upstream in the signaling network requires further investigation.

Cis-regulatory elements in promoter regions govern both spatiotemporal gene expression and responses to environmental cues. Previous studies have established that *SlCBF1, SlCBF2, SlCBF3* are key cold tolerance genes involved in plant response to low-temperature stress. Their promoter regions are enriched with various abiotic stress-related cis-acting elements, including long terminal repeat (LTR, a low-temperature-responsive element), W-box, ABRE (abscisic acid-responsive element), and STRE elements, suggesting that MdWRKY31 may directly regulate the transcription of these target genes by recognizing these elements. Together with previous findings, the regulation of downstream cold-responsive genes by MdWRKY31 may contribute to enhanced plant adaptation under cold stress [61,62]. We further predicted the interactions between the MdWRKY31 protein and the promoters of these genes using molecular docking, and the results support the above hypothesis. Nevertheless, whether MdWRKY31 specifically recognizes W-box elements in the target gene promoters to mediate transcriptional activation or repression, as well as the in vivo authenticity of these interactions, still requires further experimental validation using EMSA, ChIP-qPCR, and dual-luciferase reporter assays.

## 4. Materials and Methods

### 4.1. Plant Materials and Growth Conditions

Apple tissue-cultured seedlings and tomato plants were maintained in the tissue culture room and light-controlled growth chamber at the Facility Horticulture Engineering Center, Northeast Agricultural University. Apple tissue-cultured seedlings were grown on MS medium with 0.6 mg/L 6-BA and 0.4 mg/L IBA for 45 days, then moved to rooting medium (MS + 1.2 mg/L IBA). After 50 days, rooted plants were moved to Hoagland’s solution and cultured until reaching 8–9 fully expanded leaves. All seedlings were clonally propagated via tissue culture from the same apple genotype and originated from a single mother plant. Both wild-type and MdWRKY31-overexpressing tomato lines were cultivated in a growth substrate composed of soil, perlite, and vermiculite (2:1:1, *v*/*v*).

Healthy apple (*Malus* × *domestica* ‘Gala’) plants maintained under greenhouse conditions were used for tissue-specific expression analysis. Root, stem, leaf, flower, and fruit tissues were harvested, immediately frozen in liquid nitrogen, and stored at −80 °C until RNA extraction. For cold-stress treatment, fifteen healthy seedlings were randomly divided into five groups and exposed to 4 °C [63,64,65]. Young leaves were collected at 0, 3, 6, 12, and 24 h after treatment, immediately frozen in liquid nitrogen, and stored at −80 °C. Three independent biological replicates were analyzed for each time point, and RT-qPCR assays were performed with three technical replicates per sample [66].

### 4.2. RNA Extraction and Cloning of MdWRKY31

We extracted total RNA from the harvested plant tissues with an RNA isolation kit (Vazyme, Cat. No. RC401, Nanjing, China). First-strand cDNA was prepared with a reverse transcription kit (Vazyme, Cat. No. R323-01).

The full-length CDS of *MdWRKY31* was extracted from the apple genome database (GDDH13 v1.1). Gene-specific primers (*MdWRKY31*-F/R; Table S1) were obtained using Primer 5.0 for design. The resulting PCR products were purified with a gel extraction kit (column-based) (Sangon Biotech, Cat. No. B610353), cloned into the pEASY-T5 vector, and subsequently verified by DNA sequencing. Primer synthesis and sequencing were conducted by BGI (Beijing, China).

### 4.3. Bioinformatic Analysis of MdWRKY31

Multiple sequence alignment was carried out using DNAMAN 9.0 software. Phylogenetic analysis was performed using MEGA 7.0 software (http://www.megasoftware.net, accessed on 7 January 2025). The secondary and tertiary structures were predicted using SOPMA (https://npsa-pbil.ibcp.fr/cgi-bin/secpred_consensus.pl, accessed on 8 January 2025) and SWISS-MODEL (https://www.swissmodel.expasy.org/interactive, accessed on 8 January 2025), respectively [67]. The domain organization of the protein was ultimately characterized using the SMART tool (http://smart.embl-heidelberg.de/, accessed on 8 January 2025).

### 4.4. Subcellular Localization of MdWRKY31

The subcellular localization vector pCAMBIA1300-*MdWRKY31*::GFP was generated by inserting the CDS of *MdWRKY31* upstream of the GFP reporter gene driven by the CaMV35S promoter via homologous recombination. The recombinant plasmid was transformed into Agrobacterium tumefaciens strain GV3101 and stored at −80 °C as glycerol stocks (40% glycerol, 1:1, *v*/*v*). The construct was transiently expressed in *Nicotiana benthamiana* leaves (5 weeks old) co-expressing the nuclear marker pBI221-mCherry via Agrobacterium infiltration [68]. After 72 h, fluorescence signals were observed using a confocal laser scanning microscope (Zeiss, Oberkochen, Germany). Primers used for homologous recombination (*MdWRKY31*-F2/R2) are provided in Table S1.

### 4.5. Gene Expression Analysis of MdWRKY31

Total RNA and cDNA were prepared according to the methods outlined in Section 2.2. RT-qPCR was used to analyze the expression levels of *MdWRKY31* at different treatment time points, its expression patterns in different tissues, as well as the expression of cold-responsive genes in *MdWRKY31*-overexpressing tomato lines. Quantification of relative expression was performed using the 2^−ΔΔCT^ method [69]. Primer sequences utilized for this study are provided in Table S1.

### 4.6. Acquisition of Transgenic Tomato Materials

‘Tom’ tomato seeds were imbibed overnight in sterile distilled water, then surface-sterilized with 75% ethanol for 3 min, rinsed three times with sterile distilled water, further disinfected with 2% (*v*/*v*) sodium hypochlorite for 15 min, and washed three times with sterile distilled water. The sterilized seeds were germinated on 1/2 MS medium, and cotyledons emerged after approximately one week. The *MdWRKY31* overexpression vector was constructed using the pCAMBIA1300 and introduced into Agrobacterium tumefaciens (GV3101 Chemically Competent Cell) according to the manufacturer’s instructions (Weidi Biotechnology Co., Ltd., Shanghai, China). The bacterial culture was grown in LB medium with dual antibiotics (50 mg/L kanamycin and 100 mg/L rifampicin) at 28 °C and 200 rpm for 16 h, then resuspended in MS medium to OD_600_ 0.5 before transformation.

Tomato seedlings grown for approximately 15 days were cut into small explants using sterile scissors. Explants were pre-cultured in the dark for 48 h, then immersed in bacterial suspension with gentle shaking for 15 min. After excess bacterial suspensions were removed with sterile filter paper, explants were transferred to co-culture medium and incubated in the dark at 25 °C for 48 h. They were then placed on selection medium for resistant bud induction for approximately two weeks. Resistant buds were transferred to elongation medium until they reached about 2 cm, followed by rooting induction. Rooted plantlets were acclimatized and transplanted to soil. The formulations of all culture media used for the selection and regeneration of transgenic plants during the transformation process are shown in Table S2. Transgenic seeds were harvested and advanced to stable T2 lines through successive generations [70]. The T2 generation plants were used for stress treatments and physiological analyses.

### 4.7. Stress Treatments and Determination of Physiological Parameters in Transgenic Tomato Lines

Peroxidase (POD), superoxide dismutase (SOD), and catalase (CAT) activities were measured using the guaiacol method, the nitroblue tetrazolium (NBT) photoreduction method, and a UV spectrophotometric method, respectively [53,71,72,73,74]. Malondialdehyde (MDA) content was quantified by spectrophotometric colorimetry, while proline content was determined using the sulfosalicylic acid method [75,76]. Soluble protein content was assessed with a Bradford protein assay kit. Hydrogen peroxide (H_2_O_2_) content was measured by the ultraviolet absorption method, and superoxide anion (O_2_^−^) production was quantified via the hydroxylamine oxidation method [77,78].

### 4.8. ROS Staining in Transgenic Tomato Leaves

Five leaves of similar size were selected from wild-type (WT), empty vector (EV), and transgenic plants, and placed in separate tissue culture bottles. DAB and NBT staining were performed according to the method described by Sun et al. (2018), with observations made on the accumulation of hydrogen peroxide and O_2_^−^, respectively [79]. The experiment included three biological replicates.

### 4.9. Molecular Docking

Molecular docking simulations were performed using the AlphaFold3 website (https://alphafoldserver.com/ accessed on 7 January 2025), and visualization was carried out using PyMOL 3.1 software (PyMOL Molecular Graphics System, San Carlos, CA, USA).

### 4.10. Data Processing and Analysis

Statistical analysis was conducted with SPSS 21.0. Results are shown as mean ± SD from three replicates. One-way ANOVA and Tukey’s test were used for comparisons. Significance is indicated by * *p* ≤ 0.05 and ** *p* ≤ 0.01.

## 5. Conclusions

Cold stress severely limits apple growth and productivity. In this study, the *MdWRKY31* gene was cloned from apple (*Malus* × *domestica*), and its role in cold tolerance was systematically investigated. MdWRKY31 is a group II WRKY transcription factor localized in the nucleus, with transcript levels markedly upregulated under low-temperature treatment. Heterologous overexpression of *MdWRKY31* in tomato significantly enhanced cold tolerance, as transgenic plants exhibited reduced reactive oxygen species accumulation, increased activities of antioxidant enzymes (SOD, POD, CAT), elevated proline and soluble protein contents, and decreased malondialdehyde levels under chilling stress. Furthermore, the expression of cold-responsive genes (*SlCBF1*, *SlCBF2*, *SlCBF3*, *SlICE1*) and ABA signaling-related genes (*SlNCED1*, *SlABI5*) was substantially upregulated in transgenic lines.

The results of molecular docking simulation showed that the MdWRKY31 protein has a strong binding affinity to the W-box element in the promoter of the *SlCBF1* gene, indicating that this protein may regulate the expression of these genes through direct protein–DNA interactions. These findings indicate that *MdWRKY31* improves plant cold tolerance by CBF-dependent pathways to modulate antioxidant defenses and osmotic balance. This study provides new insights into the molecular basis of low-temperature adaptation in apple and identifies *MdWRKY31* as a promising candidate for breeding cold-resistant varieties.

## Data Availability

The data that support the findings of this study are available from the corresponding authors upon reasonable request.

## Supplementary Materials

The following supporting information can be downloaded at: https://www.mdpi.com/article/10.3390/ijms27125560/s1.
