# Supplementary figures and images for: Functional Characterization and Potential Regulatory Role of MdWRKY31 in Cold Tolerance
Source: Int J Mol Sci. 2026 Jun 19;27(12):5560. doi: 10.3390/ijms27125560 (PMC13300141; doi:10.3390/ijms27125560)

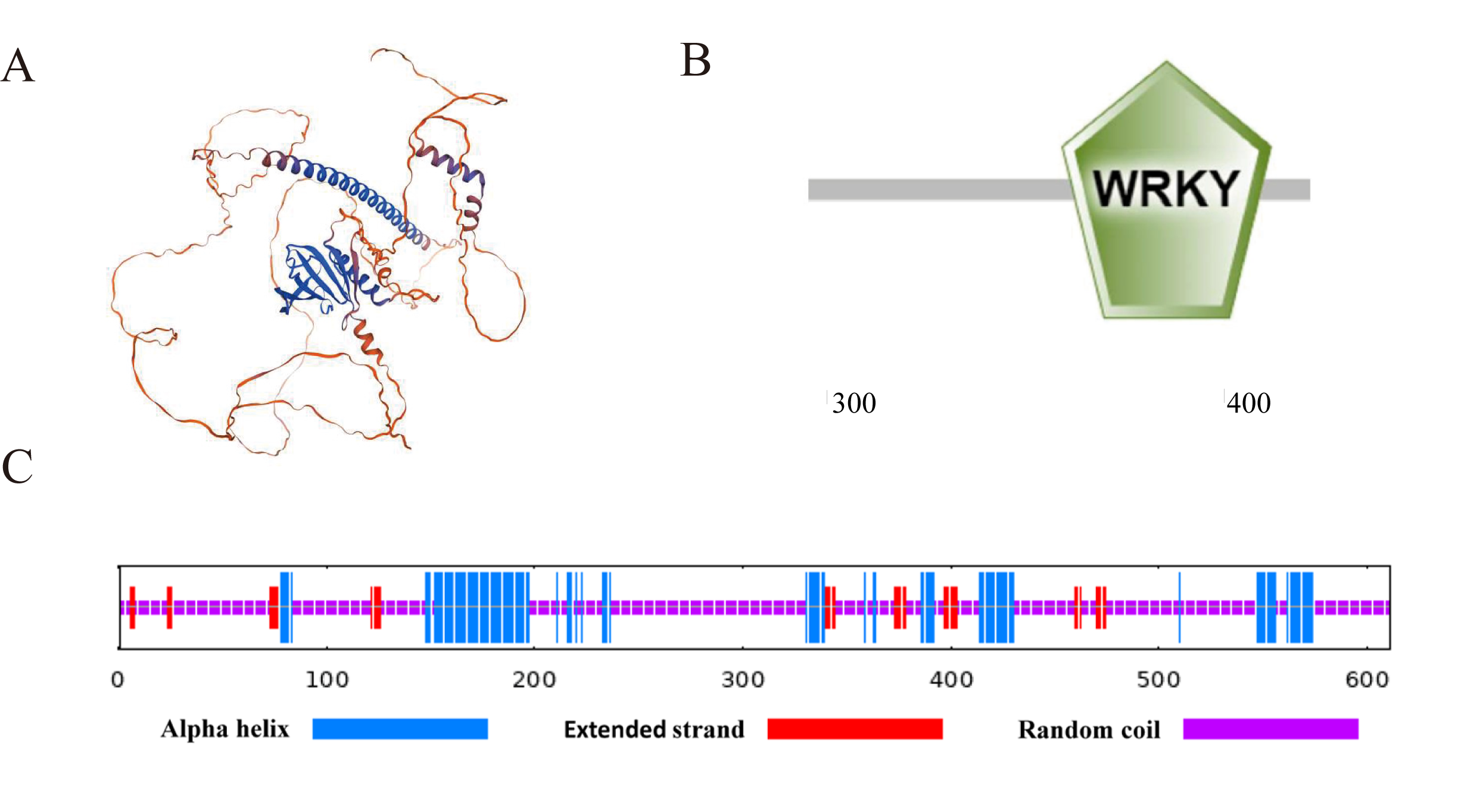

Supplement: Supplementary file 1 [file ijms-27-05560-s001.zip › Supplementary Figures/Figure S1.tif]

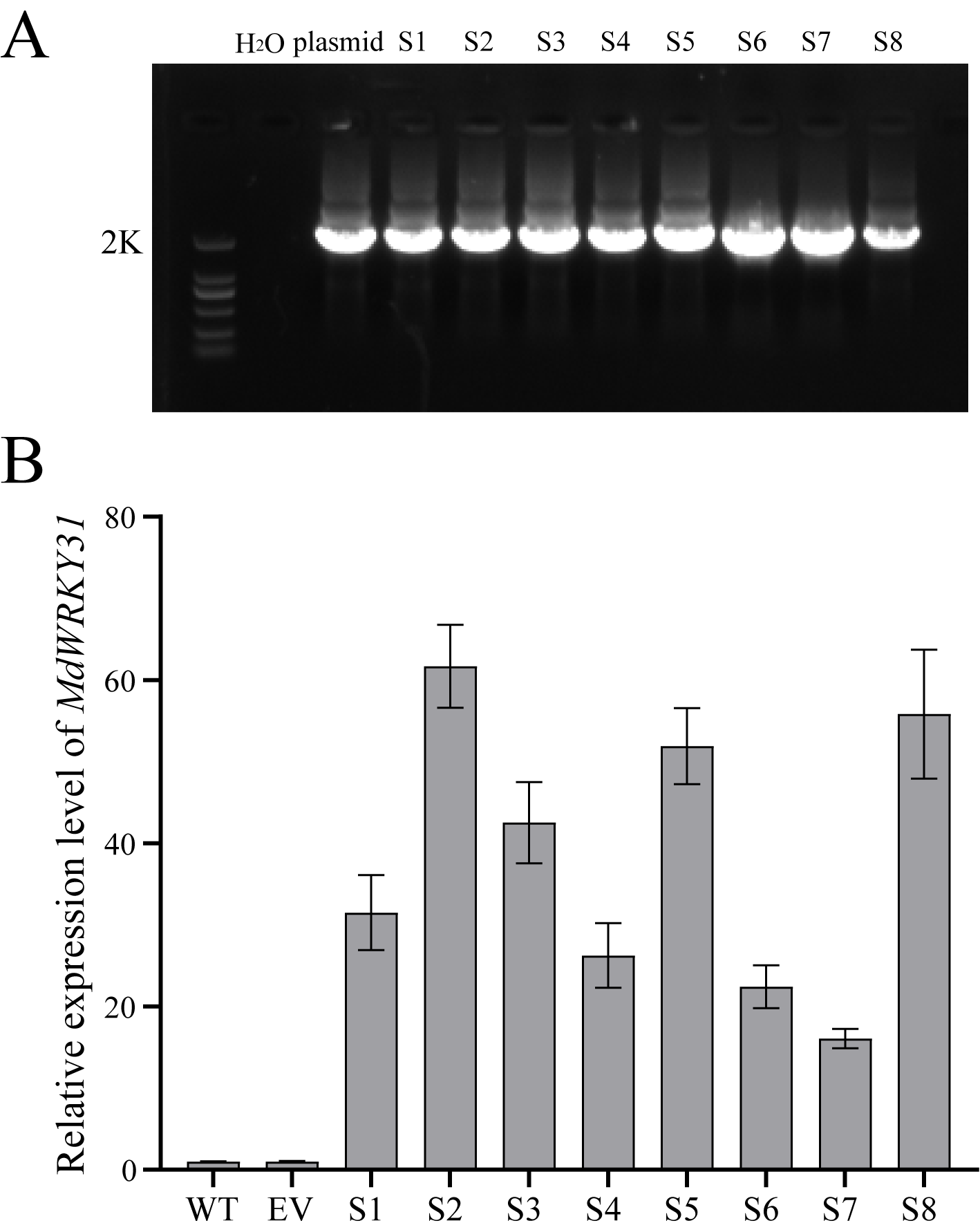

Supplement: Supplementary file 1 [file ijms-27-05560-s001.zip › Supplementary Figures/Figure S2.tif]
